# Development of Tests for Arm Coordination Impairment in Paralympic Classification

**DOI:** 10.3389/fresc.2022.865133

**Published:** 2022-07-06

**Authors:** Viola C. Altmann, Nadine Hendriks, Eline A. Lammens, Mariska Janssen

**Affiliations:** ^1^Expertise Centrum, Klimmendaal Rehabilitation Centre, Arnhem, Netherlands; ^2^Peter Harrison Centre of Disability Sport, School of Sport, Exercise and Health Sciences, Loughborough University, Loughborough, United Kingdom; ^3^World Wheelchair Rugby, Sheffield, United Kingdom; ^4^Department of Rehabilitation, Radboud University Medical Center, Donders Institute for Brain, Cognition and Behaviour, Nijmegen, Netherlands

**Keywords:** wheelchair, coordination, impairment test, wheelchair sports, Paralympic

## Abstract

**Background:**

In Paralympic sport, classification of impairment with the ability to detect misrepresentation of abilities is mandatory. In wheelchair rugby, there is currently no objective method to classify arm coordination impairment. In previous research, sufficient correlation between the spiral test (ST) and activity in wheelchair rugby was found in athletes with coordination impairment. However, the ST depends on maximum voluntary effort.

**Purpose:**

To assess if the ST is an objective test for arm coordination impairment, in which maximum voluntary effort can be distinguished from intentional misrepresentation. The aims of this study were to (1) assess the test-retest reliability of the ST and (2) assess if Fitts's law is applicable to the ST.

**Methods:**

Nineteen volunteers without impairments performed two sessions with three STs per arm. The STs were projected and measured on a tablet and had three different indices of difficulty based on differences in spiral width. The time to complete the spiral was measured and a penalty time was added for each time the borderline of the spiral was touched (3 s) or crossed (5 s).

**Results:**

Test-retest reliability was assessed using a Bland-Altman analysis and showed limits of agreement that were wider than the margins of 2SD from the group mean. Repeated measurement correlation coefficients between the index of difficulty according to Fitts's law and the movement time were > 0.95 (*p*-value < 0.001) for both test and retest. A *post-hoc* optimisation of penalty times revealed an optimum penalty time of 2.0 s for the dominant arm and 2.5 for the non-dominant arm for any contact with the margins of the spiral.

**Conclusions:**

The ST has sufficient test-retest reliability and Fitts's law is applicable. Therefore, it is a promising option for classification of arm coordination impairment with the option to distinguish intentional misrepresentation from maximum voluntary effort.

## Introduction

The Paralympic Games were founded in Great Britain after the Second World War as a sport event with the goal to enhance participation of wounded veterans in society. Over the years, sports for veterans became an international event and were connected to the Olympic Games in 1956 (1). Nowadays, the Paralympic Games are the world's third largest sports event, with athletes competing from all over the world. In 2016, broadcasting of the Paralympic Games was covered in 154 countries, with 4.1 billion people watching (2). In the early days, patients were competing against other patients with the same health condition, like Spinal Cord Injury (SCI) or amputations, in only a few sports (1). Nowadays in the Paralympic summer games, over 4,000 professional athletes compete in 22 sports and earn their daily living by it (2).

To guarantee an attractive and fair competition, the best athlete should win, and not the one who is the least impaired, and there should be enough athletes to compete against. To achieve this, athletes compete in categories (classes) in which the impact of impairment on the ability to perform should be similar. The process that leads to categorizing athletes is called classification. The aim of classification is that winning or losing the competition is based on training, motivation, talent and skills rather than the severity of impairments (3). To determine the optimal class for each athlete, testing of impairment is mandatory. However, there is a risk that athletes will try to misrepresent their abilities, to try to compete in a class with athletes with more severe impairments than their own. This is called Intentional Misrepresentation (IM). In an impairment test for classification, it should be possible to distinguish IM from Maximum Voluntary Effort (MVE). Currently, most classification systems are based on expert opinion of experienced classifiers. However, with the increasing professionalism of Paralympic sports, the International Paralympic Committee stated that classification should develop toward Evidence-Based Classification, in which the number and the borderlines of the classes per sport should be supported by empirical data (3, 4).

One of the Paralympic Sports is Wheelchair Rugby (WR). It was developed by and for athletes with tetraplegia due to spinal cord injury (SCI) in 1977. Since 2000, WR is a full medal sport in the Paralympic Games. There are more than forty countries that actively participate in WR, or who are developing WR programmes within their nation. WR was developed for athletes with SCI, but athletes with other health conditions, such as neuromuscular diseases, cerebral palsy (CP) and limb deficiencies are also allowed to compete. WR as a Paralympic sport is a mixed gender team sport with the age of elite players varying on average between 20 and 35 years. In classification in WR, athletes can be grouped in one of the seven classes: 0.5, 1.0, 1.5, 2.0, 2.5, 3.0, and 3.5 points, based on arm and trunk impairment. During competition, four athletes are on the court and the total points for one team cannot exceed eight points (5). At this moment, the impairment tests to allocate scores for arm impairment are based on muscle strength, because WR was developed for athletes with SCI. However, the number of WR athletes with coordination impairment is expected to be much higher in the future since the incidence of CP is 39–150 times as high as the incidence of SCI (6, 7). In contrast to classification of arm strength impairment and trunk impairment (including all neuromusculoskeletal impairment types), which are largely evidence based (8–13), there is very limited evidence to support the classification of arm coordination impairment (14).

None of the current classification systems in Paralympic Sports include objective, Evidence Based impairment tests for arm coordination impairment (15, 16). The spiral test (ST) is a multilevel, parsimonious tests that may be suitable for classifying arm coordination impairment in WR. In previous research, a moderate-strong correlation between the ST and activities in WR was found. Furthermore, athletes with coordination impairment could be distinguished from individuals without impairments, so that minimum impairment criteria for eligibility of arm coordination impairment could be established (14). However, the results of the ST depend on MVE and so far, it has not been assessed if IM can be distinguished from MVE in the ST. IM The next step that needs to be taken in the development of Evidence Based Classification is to assess if this distinction can be made in the ST.

In general, a method for differentiating between MVE and IM must satisfy two main criteria: (a) sufficient test-retest reliability and (b) detectable differences between the results achieved under the presence and absence of MVE. The test-retest reliability of the ST has not been assessed so far (14). To detect differences between the presence and absence of MVE, Fitts's law is a promising option (17, 18). Coordination affects both movement accuracy and movement speed. Faster movements are less accurate and higher accuracy is achieved at lower speeds (19). In Fitts's law the relationship between the movement accuracy and precision is reflected in the index of difficulty (ID). Movement time and different IDs show a significant linear relationship in tests with movements between two targets in which several target sizes and target widths result in different IDs (20). If a movement time is significantly different from that line in a minimum of three tests, this is a sign that there was no MVE in one of the tests (17). At this moment, it is not clear if Fitts's law is also applicable to the ST that was used in previous classification research in WR.

In this study we further investigated the ST as a possible objective test for arm coordination impairment in WR athletes. To elaborate the options for distinguishing MVE from IM, the aims of this study were to (1) assess the test-retest reliability of the ST and (2) assess if Fitts's law is applicable to the ST.

## Materials and Methods

### Participants

A convenience sample of nineteen adults without impairments in the same age range as athletes in Paralympic sports, (mean age of 27 years; range: 19–33), participated in this cross-sectional study. Eighty-nine percent were male (*n* = 17) and eleven percent were female (*n* = 2) similar to the ratio of men and women in wheelchair rugby (21). We selected this population with similar age and gender to WR athletes, because in previous studies there appeared to be an impact of age and gender on coordination (22). Three of the nineteen participants were left dominant, one participant was ambidextrous and fifteen participants were right dominant. Because an ambidextrous person is expected to use his right arm more than his left arm in a society with a majority of right handed persons, the ambidextrous participant's data were analyzed as right dominant. All participants gave written informed consent prior to participating, and the study was performed in accordance with the Declaration of Helsinki (2013) developed by The World Medical Association (32). The study has been assessed by the Medical Ethical Committee of the Netherlands, region Arnhem and Nijmegen, (registration number 2021-13107) and received local approval of the scientific committee of Klimmendaal rehabilitation center.

### Spiral Test

**Figure 1:**
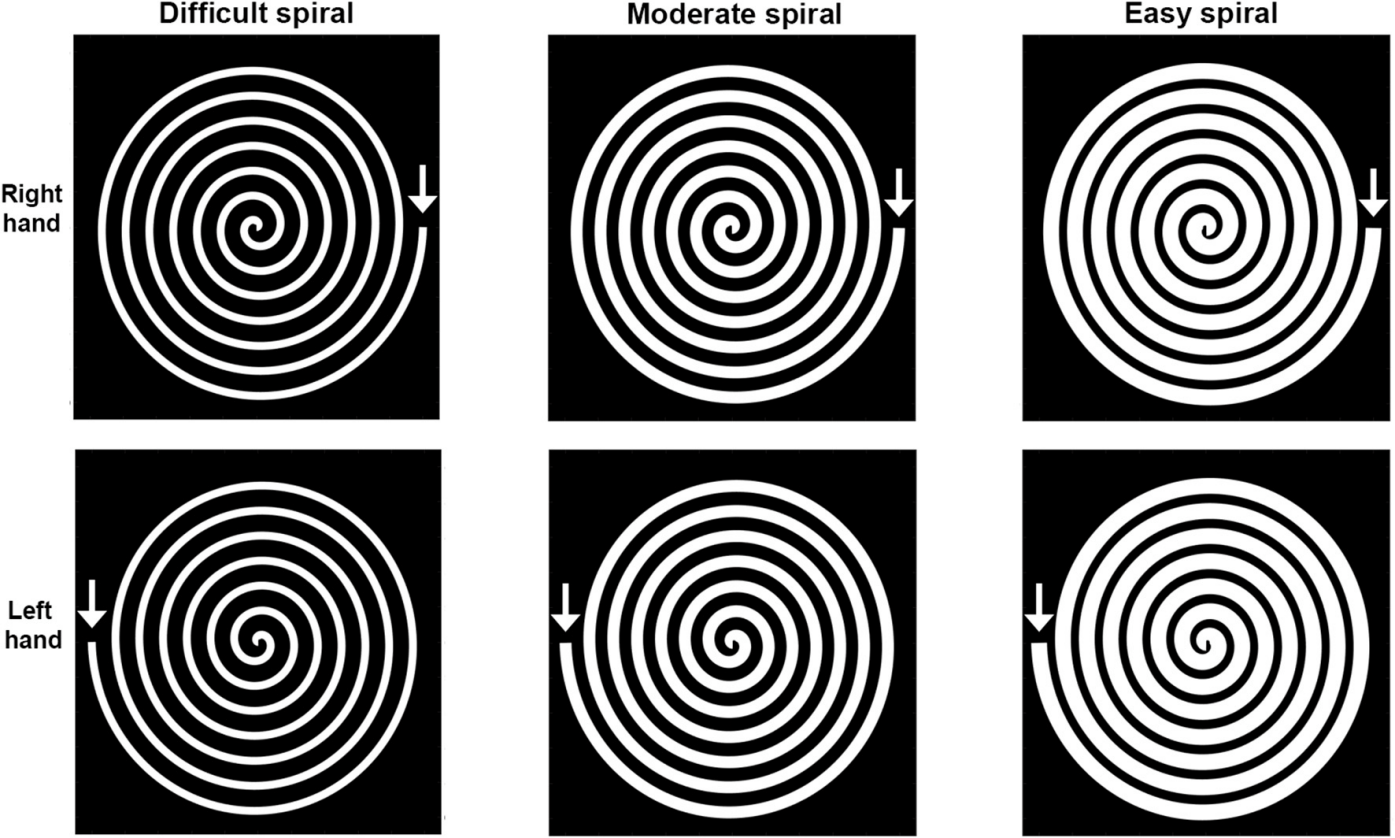
Spiral tests (ST) with different index of difficulty for the left and the right hand. Difficult (spiral width 3.5 mm), Moderate (spiral width 5.3 mm), and Easy (spiral width 7.1 mm).

All participants performed six spiral tests (STs) with a pen, three spirals with different levels of difficulty with the dominant arm and three spirals with the same, different levels of difficulty with the non-dominant arm. The level of difficulty was determined by the widths of the spirals, i.e., 3.528, 5.291, and 7.056 mm. Each spiral had seven turns with a length of 2,204,771 mm, resulting in three different IDs, i.e., difficult: 901.6, moderate: 601.1 and easy: 450.8. For each version there was a right-handed and a mirrored left-handed spiral. The right-handed spiral was completed clock-wise and the left handed counter clock-wise, see Figure 1. Spiral lengths were calculated using the function “arclength.m” in Matlab, which calculates the length of a path based on its x and y coordinates. IDs were calculated using Formula 1, where A is the length of the spiral and W is the spiral width (23).


(1) $$\begin{array}{r} Formula\;1:ID\infty =\frac{A}{W\ln\;2} \end{array}$$

This formula differs a little for the original formula for Fitts's law which was developed for a movement with only a fixed start and finish target, but a free movement trajectory between the start and the finish target. In the ST, participants had to stay the entire task with the pen within the white spiral. So besides the start and the finish, the whole movement trajectory was fixed. Figure 1 shows an example of the right- and left-handed spirals. Real size spirals are available in Supplementary Material 1.

The STs were performed on a digitized graphic tablet (Wacom Cintiq 16, model nr: DTK1660K0B, 2019) (24). Calibration of the pen was performed before the start of every measurement. Participants were asked to draw a line within the spiral as quickly and accurately as possible from the arrow to the center (Figure 1). The primary outcome measure for this test was the total time in which the spiral was completed, indicated as movement time. A 3 s penalty was added for each time the borderline between the spiral and the black area was touched with the pen, and 5 s penalty was added for each time the pen was in the black area (22). However, the spirals we used had a different lay-out with a white trajectory on a black background, compared to the original research in which the windings were in between two black lines on a white paper. Touching the lines was similar in the two lay-outs. However, crossing the line to end up in another winding was possible in the original test, but was unlikely in the lay-out we used. Therefore, we decided to use the original penalty times, but to also perform an optimisation of the penalty times for this new lay-out used in the present research.

### Test Protocol

All participants were seated in an everyday wheelchair without armrests with the brakes on while testing (Summit Benelux BV, Deventer, the Netherlands). The tablet was positioned on a height adjustable table, so the shoulder was in a neutral position and the elbow was in 90° flexion. Participants performed the three STs per arm in one session per day. The same STs were repeated on another day, 1–2 weeks apart. The order of the ST was randomized per arm and per day, so the order could be different for each arm and on each testing day. STs were recorded with a video camera and execution time was measured with a stopwatch in s.

### Data Analysis

Test-retest reliability was assessed using a Bland-Altman analysis to determine mean bias, limits of agreement (LOA) and 95%-confidence intervals (CI) of the STs. Bland-Altman analysis was used as it gives insight in both reliability and agreement of the ST which is important when the ST is going to be used in paralympic classification (25). No standard cut-off values for sufficient reliability exist in the literature for the difference between test and retest values (26). In previous research, it was possible to distinguish athletes with coordination impairment from volunteers without impairment, with a test accuracy of 93.5% using 2 standard deviations from the mean (14). Therefore, in this study STs were deemed reliable, if differences between test and retest times (i.e., limits of agreement) were within two standard deviations from the group mean. In addition, linear regression analyses were performed to examine if there was proportional bias in the data.

To test if Fitts's law was applicable to the data, we calculated repeated measure correlation coefficients (Rmcorr) between ID and movement time (27, 28).

There did not appear to be significant restriction of range or gross violations of normality based on Shapiro-Wilk test for normality and inspection of the histograms. A *post-hoc* optimisation of the penalty times was done, using a range of penalty times from 0 s to 3 s with 0.5 s intervals for both touching and crossing the lines. For this *post-hoc* optimization of we recalculated the Rmcorr for the data corrected for different penalty times for both test and retest. The penalty time with the highest Rmcorr was selected as the optimal penalty time.

## Results

**Figure 2:**
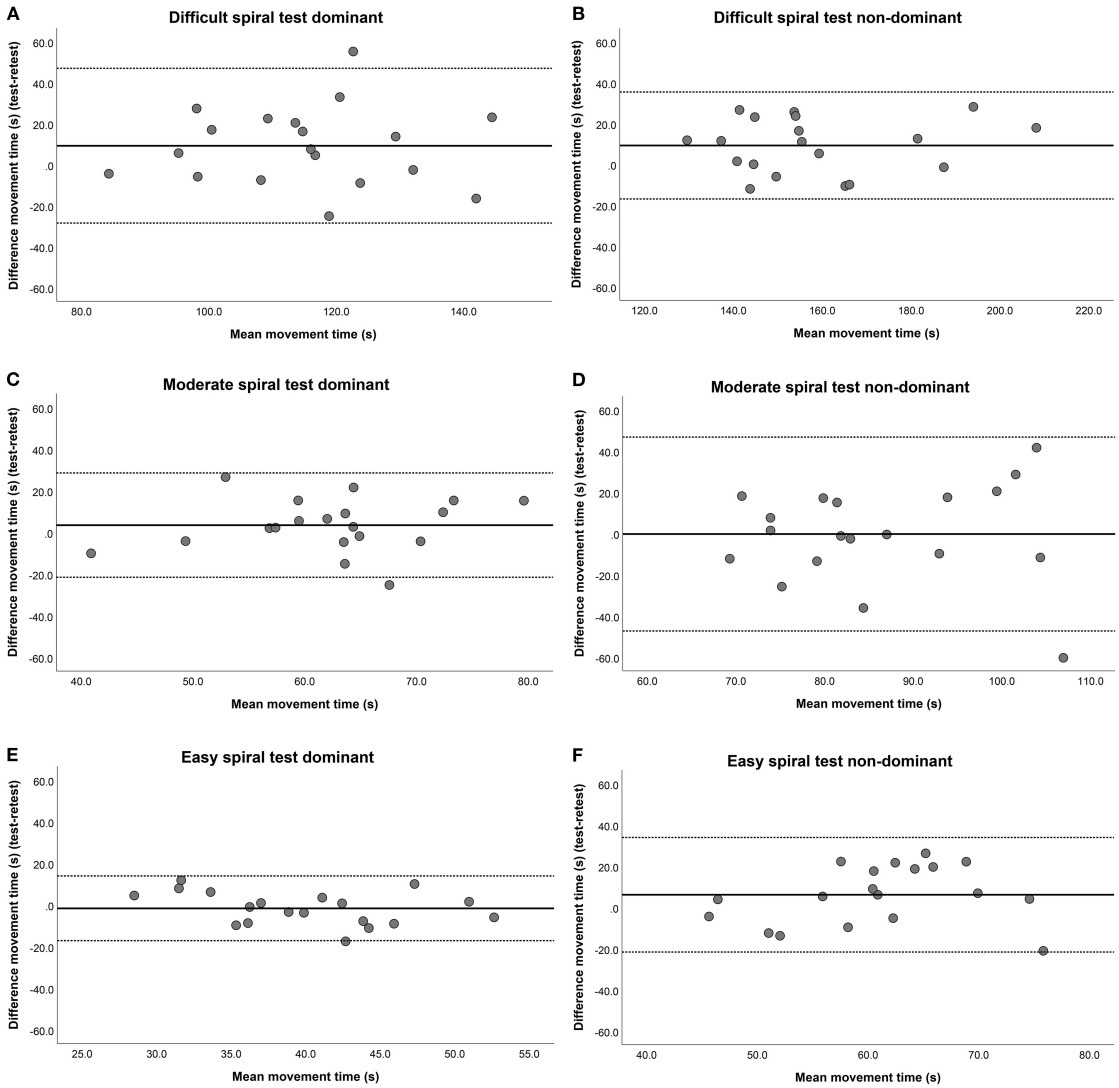
Bland-Altman plots for test-retest reliability. **(A)** ST difficult dominant side, **(B)** ST difficult non-dominant side, **(C)** ST moderate dominant side, **(D)** ST moderate non-dominant side, **(E)** ST easy dominant side and **(F)** ST easy non-dominant side. The horizontal lines represent the mean difference (Bias) and two standard deviations, i.e., LOA (dashed lines) of the differences between the movement time (s) at test and the movement time (s) at retest.

Figure 2 shows the Bland-Altman plots of all STs. On average the large spirals have the narrowest LOA. Table 1 shows the average times of test and retest per spiral, the difference between test and retest and the LOA. Most spiral tests showed no significant fixed bias, except for the difficult spiral with the non-dominant arm. That spiral had a fixed bias of 9.2 s. Furthermore, none of the spiral tests showed proportional bias. Finally, for most spiral tests the LOA were wider than the margins of 2SD of the group mean, indicating sufficient test-retest reliability, except for the most difficult spiral test with the non-dominant arm. For that test the LOA were smaller than 2SD of the group mean Table 2 shows the absolute movement time, nr. of penalties and corrected movement time for test and retest per condition. Although, on average the differences in movement time and the nr. of penalties between test and retest were small. Individually, there could large differences in (corrected) movement times and nr. of penalties, which can be seen in the outliers displayed in Figures 2A,C,D.

**Table 1:** Bland-Altman analysis for test-retest reliability.

**ST**	**Side**	***N*** **-valid**	**Test** **Mean (Sd)**	**Retest Mean (SD)**	**Group mean (test + retest)/2 Mean (SD)**	**Mean difference (LOA)**	**95% CI**	**Fixed bias**	**Regression**	***p*** **-value**	**Proportinal bias**	**LOA <2 SD group mean**
Difficult	Dominant	19	119.8 (18.6)	110.6 (18.3)	115.2 (18.8)	9.2 (−28.5 to 47.0)	(−0.1 to 18.5)	No	0.018	0.940	No	Yes
Difficult	Non-dominant	19	163.1 (22.5)	153.9 (21.2)	158.5 (22.1)	9.2 (−17.0 to 35.4)	(2.8 to 15.6)	Yes	0.103	0.676	No	No
Average	Dominant	19	64.1 (11.7)	60.7 (10.1)	62.4 (10.9)	3.4 (−21.6 to 28.5)	(−2.7 to 9.6)	No	0.159	0.516	No	Yes
Average	Non-dominant	19	86.3 (17.3)	86.6 (16.9)	86.4 (16.9)	−0.3 (−47.3 to 46.7)	(−11.9 to 11.2)	No	0.028	0.910	No	Yes
Easy	Dominant	19	39.2 (6.4)	40.8 (8.8)	40.0 (7.7)	−1.7 (−17.2 to 13.9)	(−5.5 to 2.2)	No	−0.349	0.143	No	Yes
Easy	Non-dominant	19	64.0 (12.1)	57.9 (9.8)	60.9 (11.3)	6.1 (−21.7 to 33.9)	(−0.7 to 13.0)	No	0.207	0.395	No	Yes

**Table 2:** Absolute movement time, nr. of penalties and corrected movement time for test and retest per condition.

			**Test**				**Retest**			
**ST**	**Side**	***N*** **-valid**	**Movement time**	**Nr. Penalties line touched (3 s penalty)**	**Nr. Penalties line crossed (5 s penalty)**	**Corrected movement time**	**Movement time**	**Nr. Penalties line touched (3 s penalty)**	**Nr. Penalties line crossed (5 s penalty)**	**Corrected movement time**
			**Mean (SD)**	**Mean (SD)**	**Mean (SD)**	**Mean (SD)**	**Mean (SD)**	**Mean (SD)**	**Mean (SD)**	**Mean (SD)**
Difficult	Dominant	19	53.2 (18.9)	15 (6)	4 (4)	119.8 (18.6)	47.9 (13.7)	15 (4)	4 (3)	110.6 (18.3)
Difficult	Non-dominant	19	61.5 (24.2)	16 (5)	10 (5)	163.1 (22.5)	57.2 (18.4)	18 (5)	9 (4)	153.9 (21.2)
Average	Dominant	19	37.8 (12.2)	6 (4)	2 (1)	64.1 (11.7)	36.4 (9.2)	6 (2)	1 (2)	60.7 (10.1)
Average	Non-dominant	19	46.3 (17.4)	6 (4)	4 (3)	86.3 (17.3)	42.8 (11.9)	7 (3)	5 (3)	86.6 (16.9)
Easy	Dominant	19	30.1 (7.8)	2 (1)	1 (1)	39.2 (6.4)	30.4 (7.5)	2 (2)	1 (1)	40.8 (8.8)
Easy	Non-dominant	19	36.3 (10.9)	5 (2)	3 (2)	64.0 (12.1)	35.6 (8.0)	4 (3)	2 (2)	57.9 (9.8)

**Figure 3:**
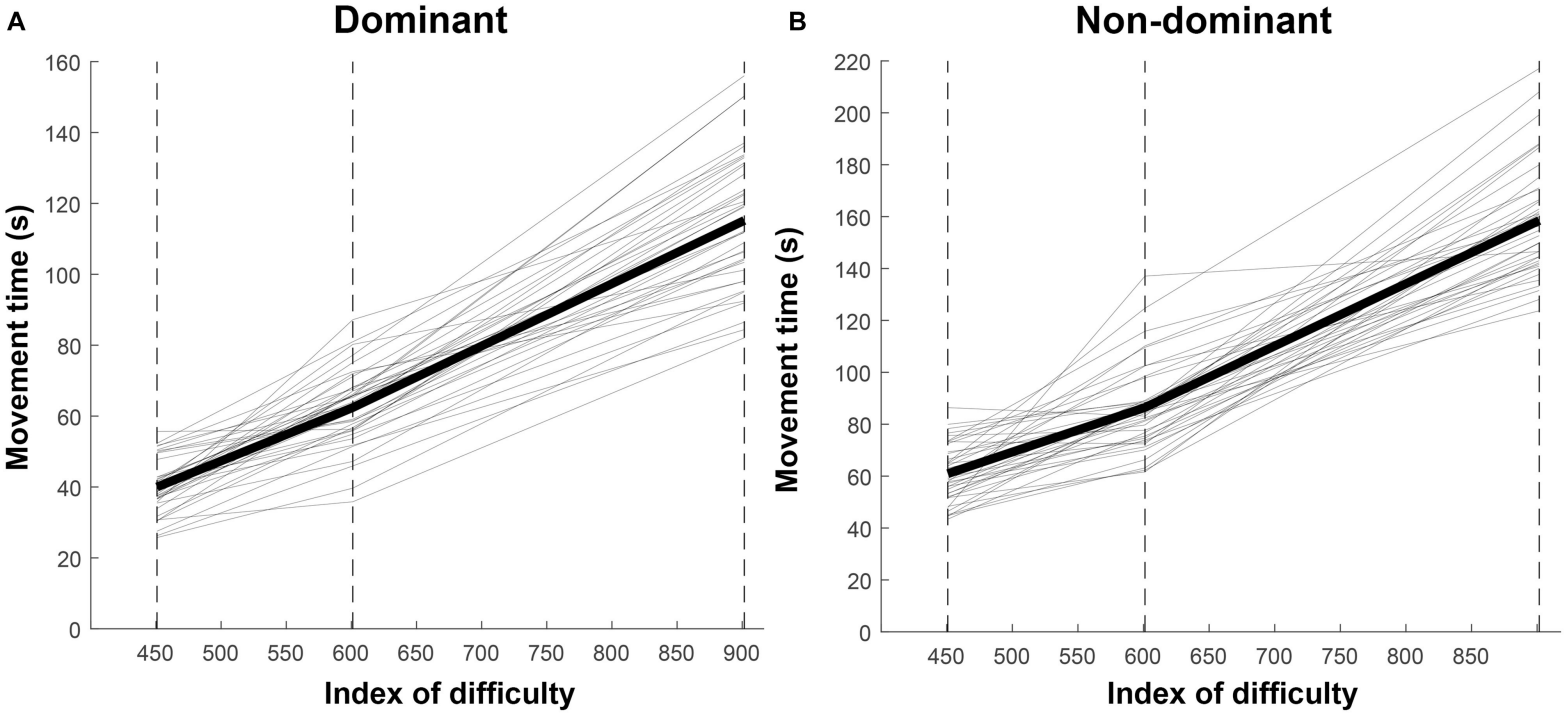
Line graph of movement time relative to index of difficulty. **(A)** dominant side and **(B)** non-dominant side. The thin lines show the participants individual results and the thick line shows the average over all participants. The dashed lines show the *x*-position of the measured indices of difficulty.

Regarding the applicability of Fitts law to the spiral test, the Rmcorr between ID and movement time was 0.97 (*p*-value < 0.001) for the test at the dominant side and 0.96 (*p*-value < 0.001) for the retest at the dominant side. For the non-dominant side Rmcor was 0.95 (*p*-value < 0.001) for the test and 0.95 (*p*-value < 0.001) for the retest. Indicating that Fitts' law is applicable to the data. Figure 3 shows the relationship between ID and movement time for the dominant and non-dominant side. Both individual data and the average are shown.

Table 3 shows the *post-hoc* optimisation of the penalty times, which showed the best fit to Fitts's law for 2.5 s for the dominant arm and 2 s for the non-dominant arm for both touching and crossing the black area with the pen.

**Table 3:** *Post-hoc* analysis for penalty time optimization.

	**Test**	**Retest**
**Penalty time (s)**	**Rmcorr**	**Rmcorr**
**Dominant**		
0	0.83	0.85
0.5	0.92	0.92
1	0.96	0.95
1.5	0.97	0.96
2	0.98	0.96
2.5	0.98^*^	0.96
3	0.98	0.96^*^
Original^†^	0.97	0.96
**Non-dominant**		
0	0.86	0.85
0.5	0.92	0.92
1	0.95	0.94
1.5	0.95	0.95
2	0.98^*^	0.96^*^
2.5	0.96	0.95
3	0.95	0.96
Original^†^	0.95	0.95

† *Original penalty time of 3 s for touching the line and 5 s for crossing the line*.

* *Highest Rmcorr between index of difficulty and movement time*.

## Discussion

In the present study, we assessed if the ST is an objective test that can be used in classification of arm coordination impairment in WR in which MVE can be distinguished from IM. To make this distinction, the ST should have sufficient test-retest reliability and Fitts's law should apply to a minimum of three spirals with different IDs. We found sufficient test-retest reliability, using a Bland-Altman analysis with a cut-off in the limits of agreement of 2 SD from the group mean between the two individual attempts for each spiral width. Only the difficult spiral test with the non-dominant arm did not meet this criterion, which was caused by the large variation in number of penalties (SD for combined 3 and 5 s. penalties more than 9) that resulted in a large variation in movement times between two attempts (SD = 22,5 s. for the test and 21.2 s for the retest). Furthermore, Fitts's law could be applied if three spirals with different IDs were used. Optimisation of the penalty times that were added to the movement time in case the borderlines of the spiral were hit or crossed, resulted in a better fit to Fitts's law.

Although the test-retest reliability was sufficient, there was a tendency for a larger variation between the two attempts if the ID was higher, except for the small spiral width (highest ID) for the non-dominant arm. This was reflected in more variation (i.e., larger limits of agreement) between the two attempts in the small spiral width (higher ID), than in the spiral with the large spiral width (lower ID). The exception for the ST with the highest ID in the non-dominant arm was caused by a combination of a slow movement and a high number of penalties, which resulted in some trade-off in the total movement time between the test and the retest. In athletes with coordination impairment, we expect a longer movement time and more penalties, which could potentially result in a decrease of the test-retest reliability which is not acceptable. However, all spirals used had a very high ID (450.8–901.6) compared to previous research in arm coordination impairment using Fitts's law (3–5) in which tapping tests were used (17). So there is more than enough room to decrease the ID by decreasing the number of windings, which will increase the test-retest reliability, so it will also be acceptable in athletes with coordination impairment. Therefore, we advise to use spirals with fewer windings than the current seven turns to increase test-retest reliability. The optimum number of windings still needs to be determined in additional research.

In the spirals with the highest IDs, the longer movement time and the variation in movement time was not only caused by slower movements, but also by more often touching or crossing the black area resulting in more penalty time. This may be a sign that the penalty time used in the original research of the ST is too long (22). This was one of the reasons we performed a *post-hoc* optimisation of the penalty times. There was also a difference in variation between the two attempts in the dominant vs. the non-dominant arm, in which it took generally longer to complete the spiral with the non-dominant arm and there was more variation in movement time with the non-dominant arm. Again, the longer movement times were for a large part determined by the penalty times that were the same for the dominant and the non-dominant arm. In previous research, the final position accuracy of the movement in a reaching task was similar in the dominant and the non-dominant arm. However, the movement trajectory was different, with a longer trajectory for the non-dominant arm (29). In the ST in which the trajectory is restricted, this can result in a longer movement time and/or more penalty time for the non-dominant arm than for the dominant arm. This was a second reason to optimize the penalty times and to consider a difference in penalty time between the dominant and the non-dominant arm. The final reason for *post-hoc* optimisation of the penalty times was the difference in lay-out of the ST used in the present research, compared to the ST in the original research. In the present research, the difference in penalty time between touching and crossing the line of the spiral width seemed less relevant, because crossing the black area with the pen to end up in the next winding did not occur. Based on the *post-hoc* optimisation of the penalty times, we found the highest Rmcorr with a penalty time of 2.5 s for the dominant arm and 2.0 s for the non-dominant arm for any contact with the black surface. In addition to lowering the number of spiral widths, we advise to optimize the penalty time into one penalty time for any contact with the black surface, but separately for the arms, 2.5 s for the dominant arm and 2.0 s for the non-dominant arm.

The applicability of Fitts's law for the ST is promising to detect IM. But to be a valid method for differentiating between MVE and IM two main criteria must be met: (1) there must be significant differences between the results achieved under MVE and IM conditions; and (2) there must be acceptable sensitivity and specificity (17). The penalties for IM during classification are severe, ranging from banning from the competition where the IM occurred to a lifetime ban for all Paralympic sports. Besides, there are potential substantial ethical and legal consequences for labeling an athlete as a cheat (30). Therefore, maximum specificity for detecting IM is crucial, to avoid false accusations. However, sensitivity must be high enough, to discourage athletes to attempt IM (31). A threshold for deviation from the line of Fitts's law to label the test result as IM with close to perfect specificity and optimal sensitivity still needs to be determined.

### Strengths and Limitations

The strength of this study is that the participants were volunteers with the same age and gender as WR athletes. This match was chosen, because there is an impact of age and gender on coordination (22). Because of the match, the study results can be used as a reference/normal values for future research in athletes with coordination impairment. Another strength is that the ST was performed on a tablet instead of on paper like in previous research (14). It will be easier to make more than the minimum of three STs with different IDs to increase the precision of the application of Fitts's law, which can enhance the sensitivity and the specificity for IM.

More difficult versions of the ST with longer movement times, resulted in more variation of the MT. We anticipate that athletes with arm coordination impairment will need longer MT to complete even easier versions of the ST. In future research in the ST in athletes with arm coordination impairments the optimal ID (spiral length/number of windings and spiral width) need to be determined for maximum reliability and the applicability of Fitts's law. In addition, we would like to collect objective tracking data from the pen and tablet (i.e., movement time, x- and y-coordinates and pen pressure) for better accuracy of the movement time and to determine if these parameters could give additional insight in intentional misrepresentation.

The present study is only focussing on developing optimal tests for arm coordination impairment. If the spiral test is an optimal and parsimonious test for arm coordination impairment, assessment in athletes with an underlying health condition that leads to coordination impairment will be the next step. This research should include the assessment of the relationship between test outcomes and performance in standardized activities that determine proficiency in WR. Only after finalizing these additional steps, evidence-based classification can be achieved.

## Conclusions

The ST is a parsimonious test that provides an objective, reliable, compound measure for coordination impairment at all joint levels of the arm. Furthermore, it is a feasible test that requires minimum equipment (14). The current research provides supporting evidence that IM may also be detected successfully using the ST. These features are promising for future use in classification of arm coordination impairment in Paralympic sports such as WR.

## Data Availability Statement

The raw data supporting the conclusions of this article will be made available by the authors, without undue reservation.

## Ethics Statement

The studies involving human participants were reviewed and approved by Medical Ethical Committee of the Netherlands, region Arnhem and Nijmegen. The patients/participants provided their written informed consent to participate in this study.

## Author Contributions

VA, NH, EL, and MJ formulated the research question and they established the study design and discussed the study results and contributed to the manuscript. NH, MJ, and VA performed the measurements and the data analysis. All authors contributed to the article and approved the submitted version.

## Conflict of Interest

The authors declare that the research was conducted in the absence of any commercial or financial relationships that could be construed as a potential conflict of interest. The reviewer MH declared a shared affiliation with the author VA to the handling editor at time of review.

## Publisher's Note

All claims expressed in this article are solely those of the authors and do not necessarily represent those of their affiliated organizations, or those of the publisher, the editors and the reviewers. Any product that may be evaluated in this article, or claim that may be made by its manufacturer, is not guaranteed or endorsed by the publisher.
