# Supplementary material for: Development of Tests for Arm Coordination Impairment in Paralympic Classification
Source: Front Rehabil Sci. 2022 Jul 6;3:865133. doi: 10.3389/fresc.2022.865133 (PMC9397744; doi:10.3389/fresc.2022.865133)

**Attachment 1: Spirals used real size**

Easy spiral – right hand


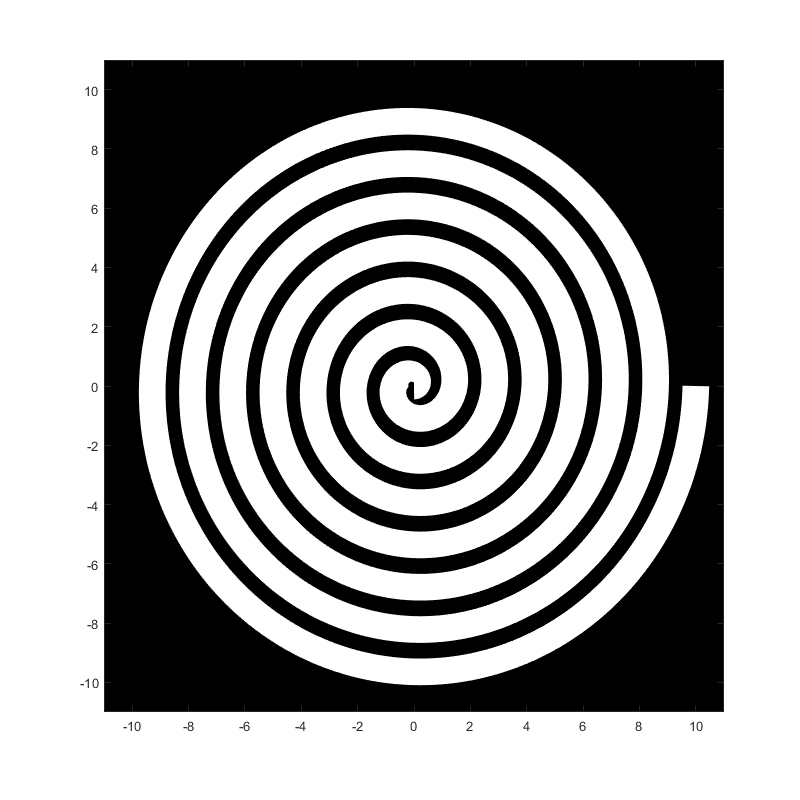


Easy spiral – left hand

Moderate spiral – right hand


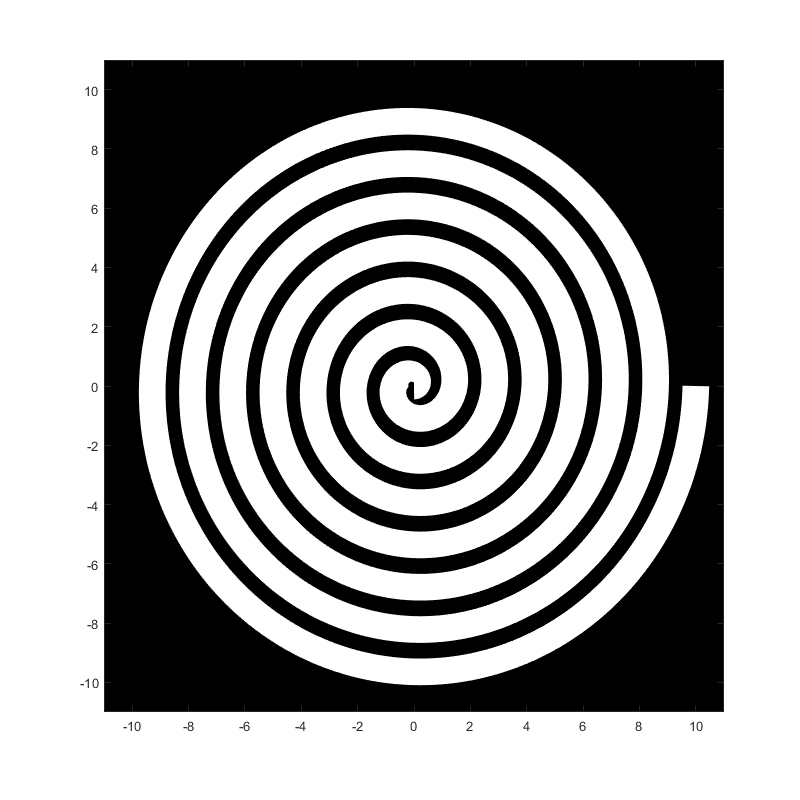

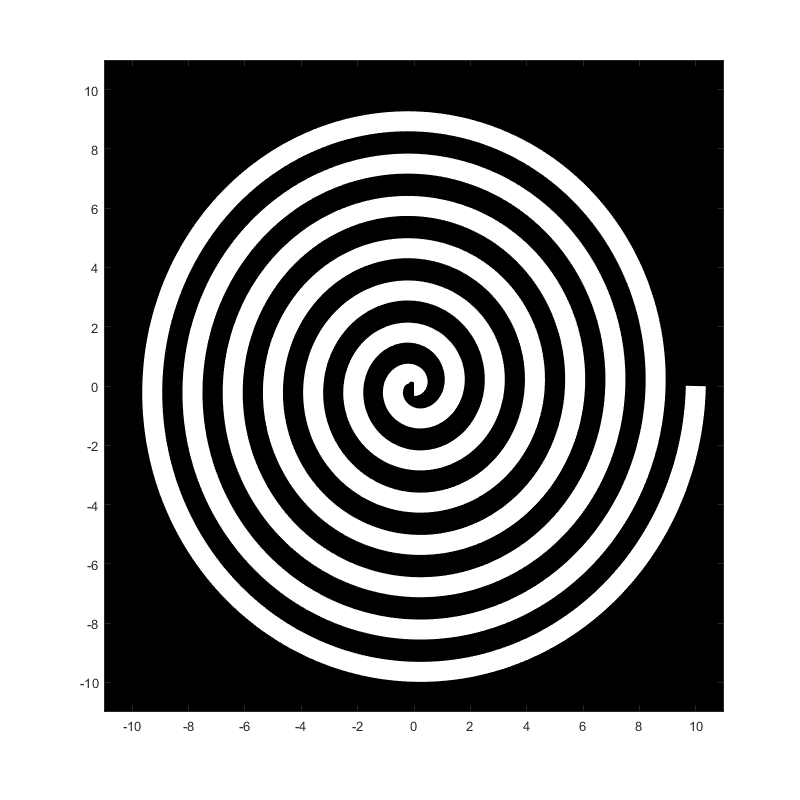


Moderate spiral – left hand


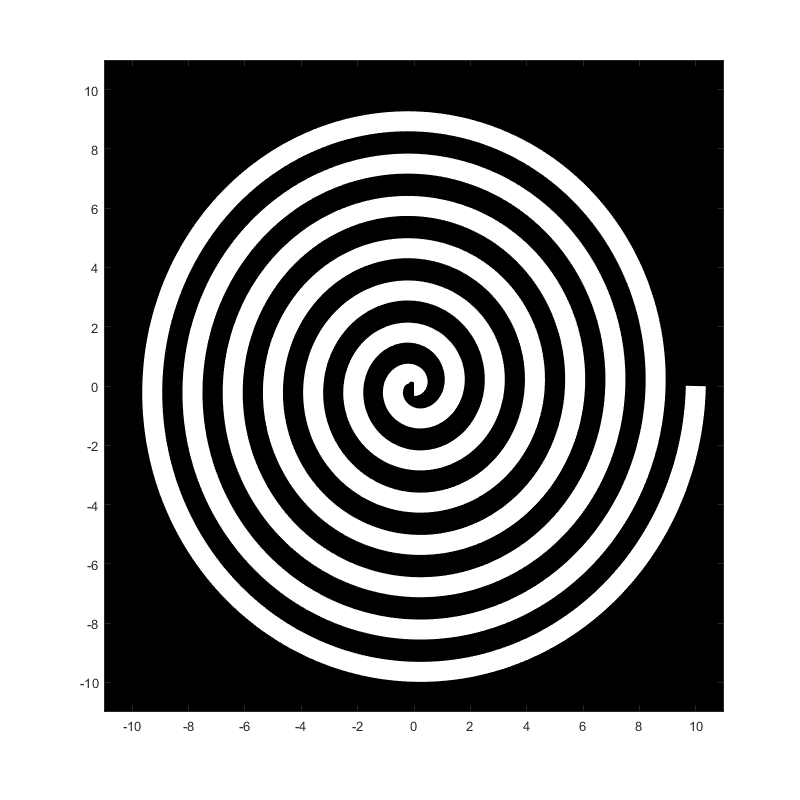


Difficult spiral – right hand


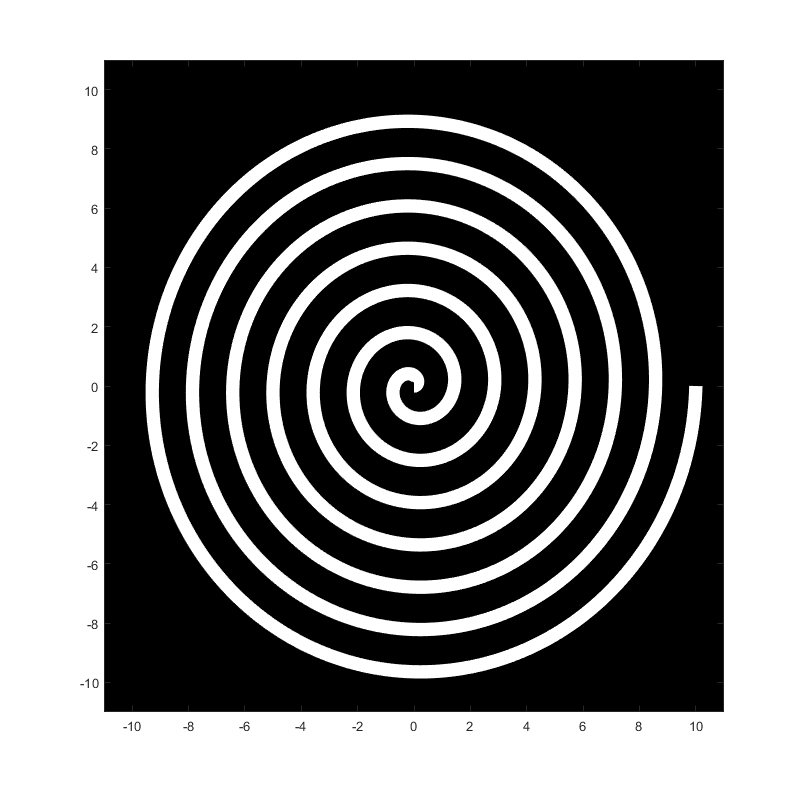


Difficult spiral – left hand


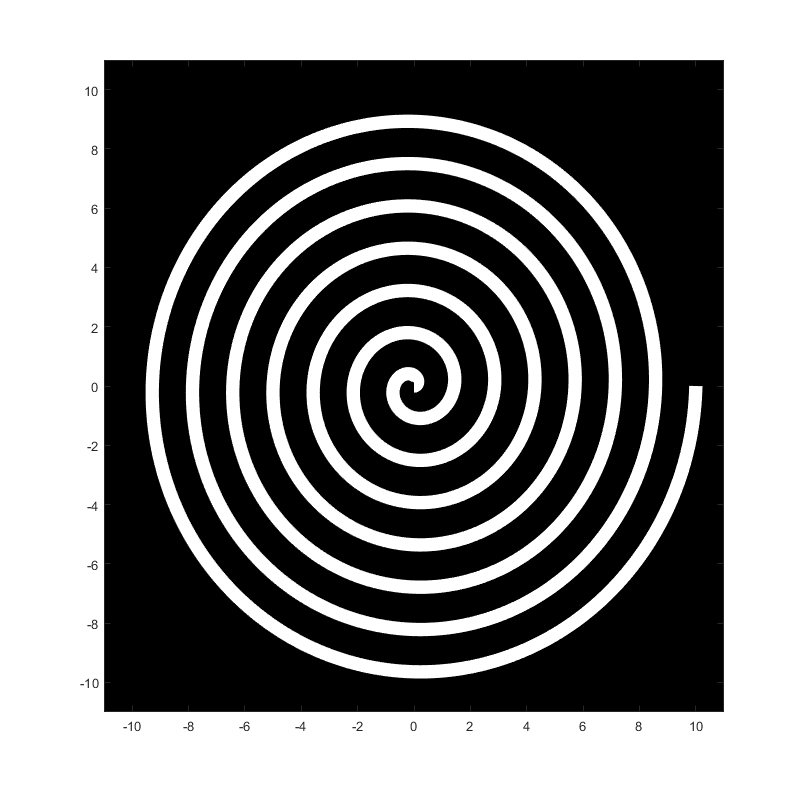

Supplement: Supplementary file 1 [file Data_Sheet_1.DOCX]
